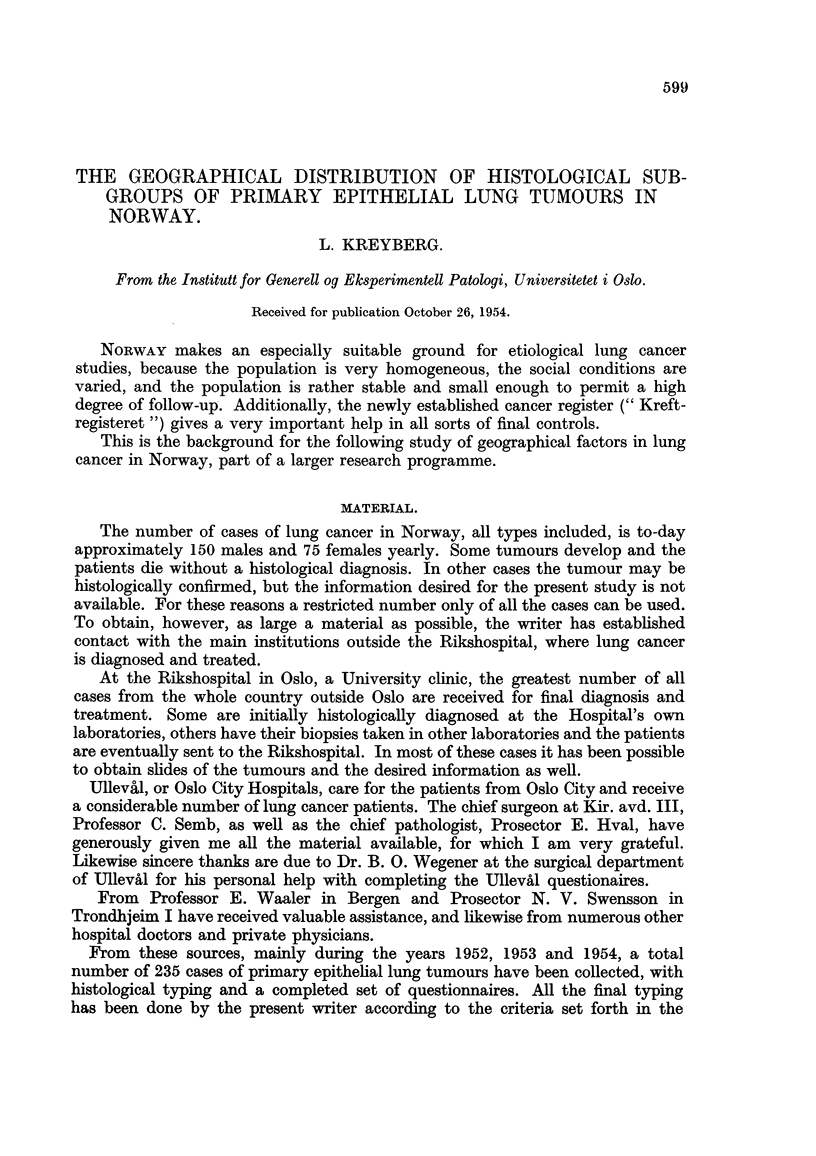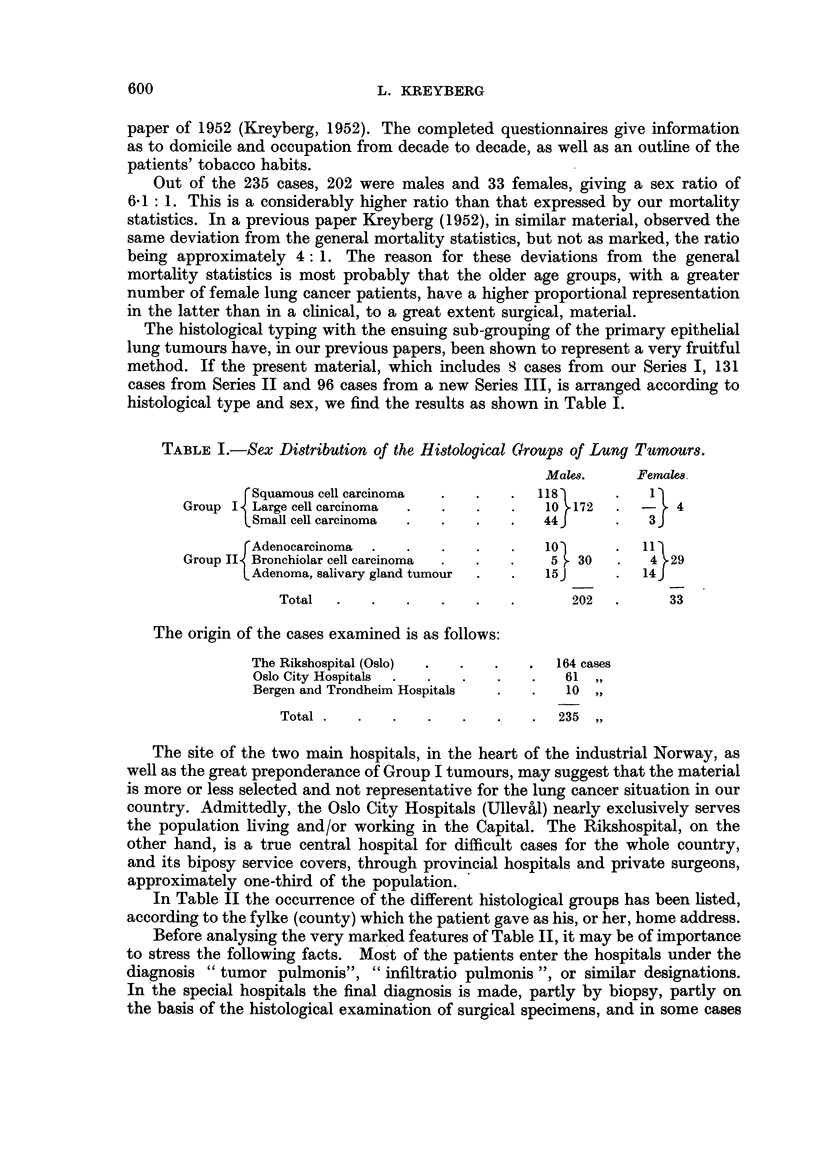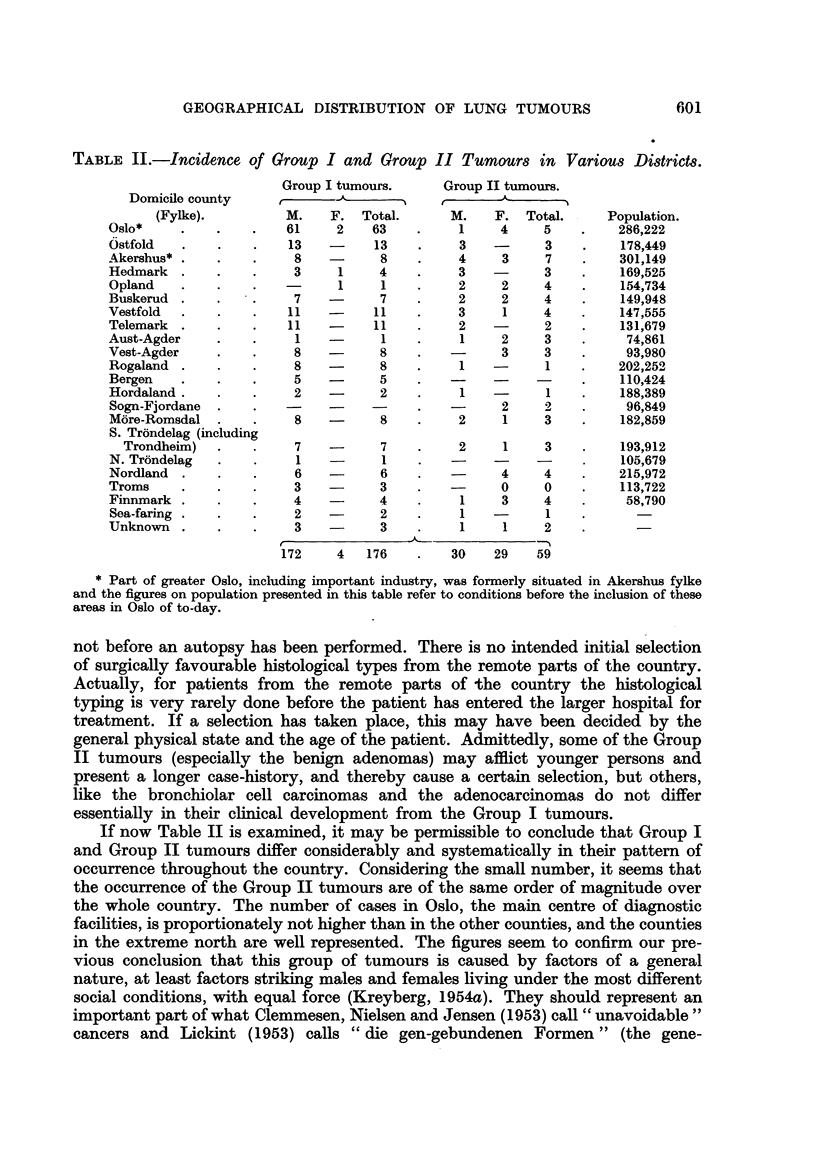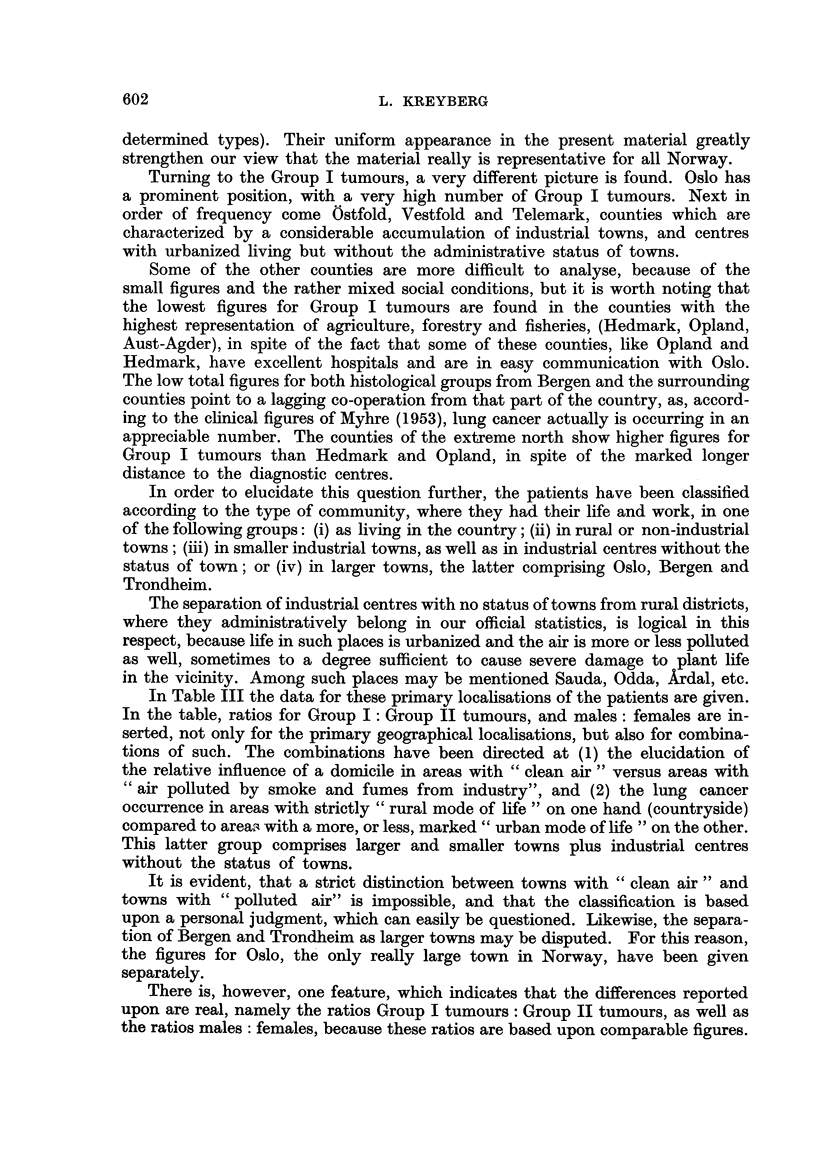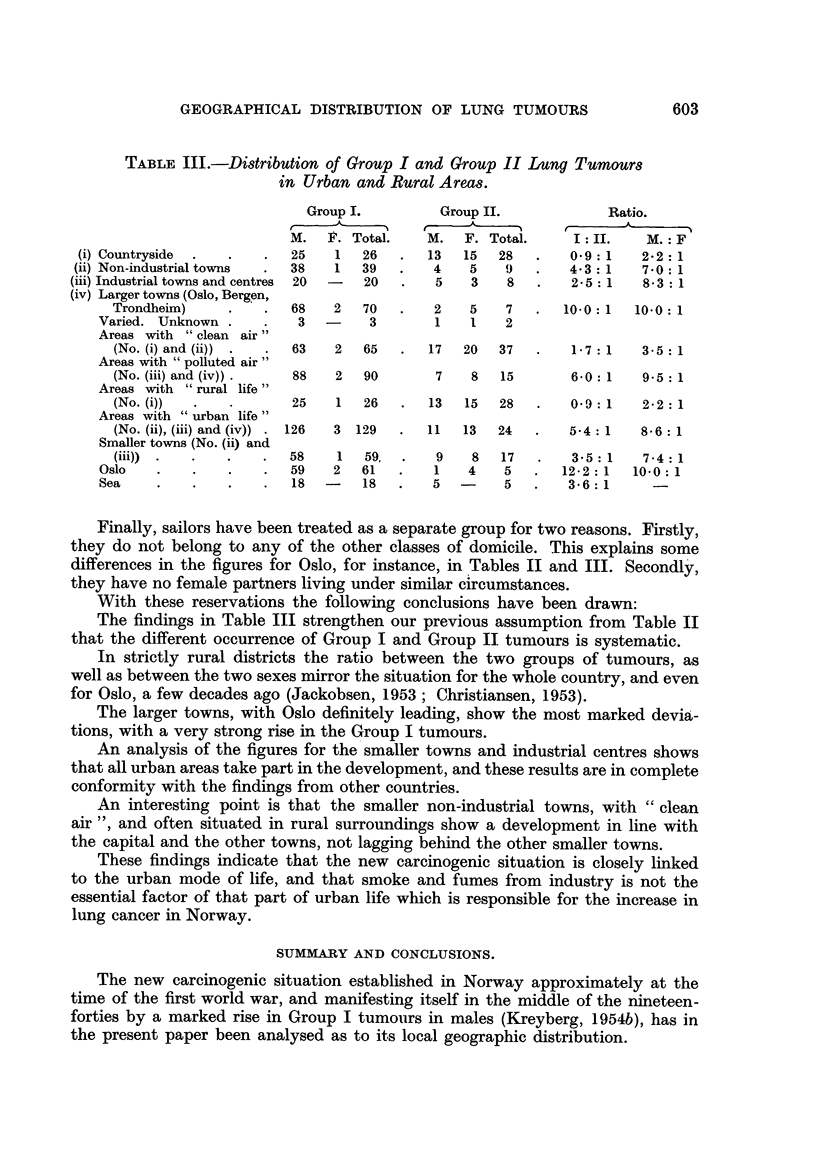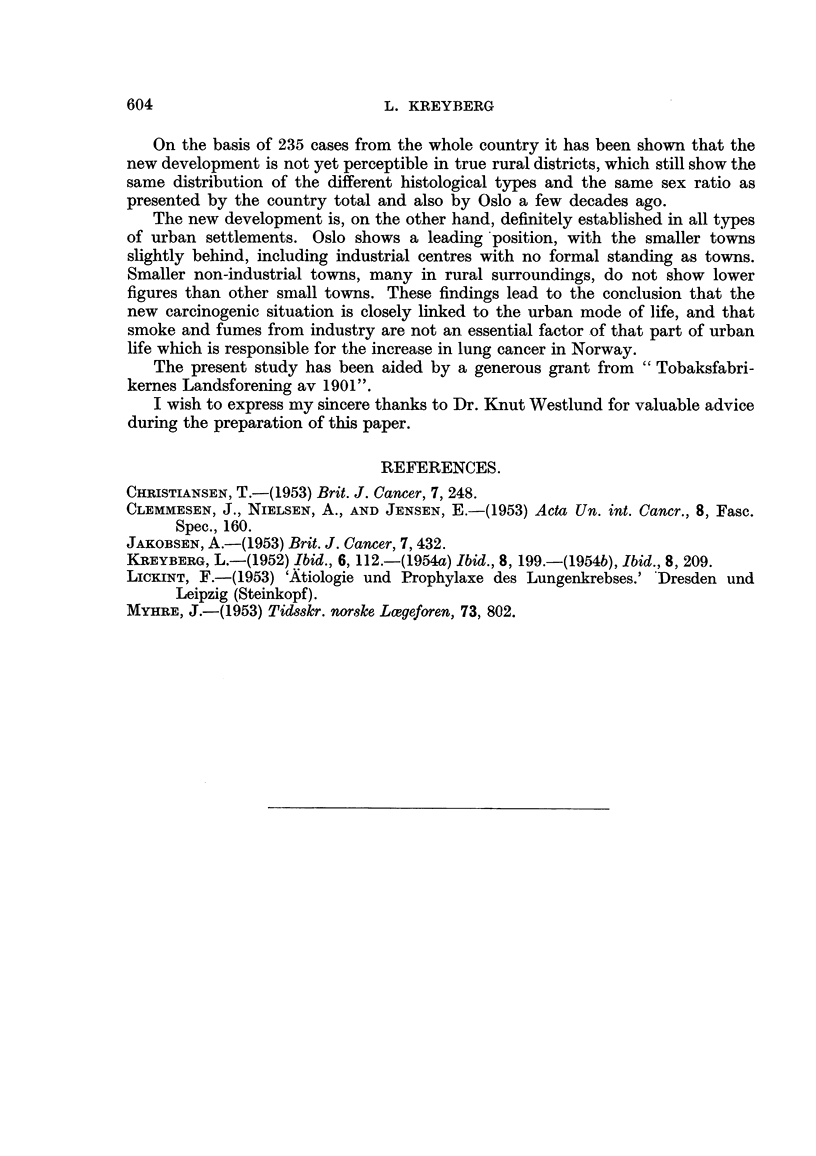# The Geographical Distribution of Histological Sub-groups of Primary Epithelial Lung Tumours in Norway

**DOI:** 10.1038/bjc.1954.65

**Published:** 1954-12

**Authors:** L. Kreyberg


### THE GEOGRAPHICAL DISTRIBUTION OF HISTOLOGICAL SUB-GROUPS OF PRIMARY EPITHELIAL LUNG TUMOURS IN NORWAY.

L. KREYBERG.

*From the Institutt for Generell og Eksperimentell Patologi, Universitetet i Oslo.*

Received for publication October 26, 1954.

NORWAY makes an especially suitable ground for etiological lung cancer studies, because the population is very homogeneous, the social conditions are varied, and the population is rather stable and small enough to permit a high degree of follow-up. Additionally, the newly established cancer register (" Kreft-registeret ") gives a very important help in all sorts of final controls.

This is the background for the following study of geographical factors in lung cancer in Norway, part of a larger research programme.

### MATERIAL.

The number of cases of lung cancer in Norway, all types included, is to-day approximately 150 males and 75 females yearly. Some tumours develop and the patients die without a histological diagnosis. In other cases the tumour may be histologically confirmed, but the information desired for the present study is not available. For these reasons a restricted number only of all the cases can be used. To obtain, however, as large a material as possible, the writer has established contact with the main institutions outside the Rikshospital, where lung cancer is diagnosed and treated.

At the Rikshospital in Oslo, a University clinic, the greatest number of all cases from the whole country outside Oslo are received for final diagnosis and treatment. Some are initially histologically diagnosed at the Hospital's own laboratories, others have their biopsies taken in other laboratories and the patients are eventually sent to the Rikshospital. In most of these cases it has been possible to obtain slides of the tumours and the desired information as well.

Ullevål, or Oslo City Hospitals, care for the patients from Oslo City and receive a considerable number of lung cancer patients. The chief surgeon at Kir. avd. III, Professor C. Semb, as well as the chief pathologist, Prosector E. Hval, have generously given me all the material available, for which I am very grateful. Likewise sincere thanks are due to Dr. B. O. Wegener at the surgical department of Ullevål for his personal help with completing the Ullevål questionaires.

From Professor E. Waaler in Bergen and Prosector N. V. Swensson in Trondhjeim I have received valuable assistance, and likewise from numerous other hospital doctors and private physicians.

From these sources, mainly during the years 1952, 1953 and 1954, a total number of 235 cases of primary epithelial lung tumours have been collected, with histological typing and a completed set of questionnaires. All the final typing has been done by the present writer according to the criteria set forth in the paper of 1952 (Kreyberg, 1952). The completed questionnaires give information as to domicile and occupation from decade to decade, as well as an outline of the patients' tobacco habits.

Out of the 235 cases, 202 were males and 33 females, giving a sex ratio of 6·1 : 1. This is a considerably higher ratio than that expressed by our mortality statistics. In a previous paper Kreyberg (1952), in similar material, observed the same deviation from the general mortality statistics, but not as marked, the ratio being approximately 4 : 1. The reason for these deviations from the general mortality statistics is most probably that the older age groups, with a greater number of female lung cancer patients, have a higher proportional representation in the latter than in a clinical, to a great extent surgical, material.

The histological typing with the ensuing sub-grouping of the primary epithelial lung tumours have, in our previous papers, been shown to represent a very fruitful method. If the present material, which includes 8 cases from our Series I, 131 cases from Series II and 96 cases from a new Series III, is arranged according to histological type and sex, we find the results as shown in Table I.

TABLE I.—*Sex Distribution of the Histological Groups of Lung Tumours.*

| | | *Males.* | | *Females.* | |
|---|---|---|---|---|---|
| Group I | Squamous cell carcinoma | 118 | 172 | 1 | 4 |
| | Large cell carcinoma | 10 | | — | |
| | Small cell carcinoma | 44 | | 3 | |
| Group II | Adenocarcinoma | 10 | 30 | 11 | 29 |
| | Bronchiolar cell carcinoma | 5 | | 4 | |
| | Adenoma, salivary gland tumour | 15 | | 14 | |
| | Total | | 202 | | 33 |

The origin of the cases examined is as follows:

| | |
|---|---|
| The Rikshospital (Oslo) | 164 cases |
| Oslo City Hospitals | 61 „ |
| Bergen and Trondheim Hospitals | 10 „ |
| Total | 235 „ |

The site of the two main hospitals, in the heart of the industrial Norway, as well as the great preponderance of Group I tumours, may suggest that the material is more or less selected and not representative for the lung cancer situation in our country. Admittedly, the Oslo City Hospitals (Ullevål) nearly exclusively serves the population living and/or working in the Capital. The Rikshospital, on the other hand, is a true central hospital for difficult cases for the whole country, and its biposy service covers, through provincial hospitals and private surgeons, approximately one-third of the population.

In Table II the occurrence of the different histological groups has been listed, according to the fylke (county) which the patient gave as his, or her, home address.

Before analysing the very marked features of Table II, it may be of importance to stress the following facts. Most of the patients enter the hospitals under the diagnosis "tumor pulmonis", "infiltratio pulmonis", or similar designations. In the special hospitals the final diagnosis is made, partly by biopsy, partly on the basis of the histological examination of surgical specimens, and in some cases

TABLE II.—*Incidence of Group I and Group II Tumours in Various Districts.*

| Domicile county (Fylke). | Group I tumours. M. | F. | Total. | Group II tumours. M. | F. | Total. | Population. |
|---|---|---|---|---|---|---|---|
| Oslo* | 61 | 2 | 63 | 1 | 4 | 5 | 286,222 |
| Östfold | 13 | — | 13 | 3 | — | 3 | 178,449 |
| Akershus* | 8 | — | 8 | 4 | 3 | 7 | 301,149 |
| Hedmark | 3 | 1 | 4 | 3 | — | 3 | 169,525 |
| Opland | — | 1 | 1 | 2 | 2 | 4 | 154,734 |
| Buskerud | 7 | — | 7 | 2 | 2 | 4 | 149,948 |
| Vestfold | 11 | — | 11 | 3 | 1 | 4 | 147,555 |
| Telemark | 11 | — | 11 | 2 | — | 2 | 131,679 |
| Aust-Agder | 1 | — | 1 | 1 | 2 | 3 | 74,861 |
| Vest-Agder | 8 | — | 8 | — | 3 | 3 | 93,980 |
| Rogaland | 8 | — | 8 | 1 | — | 1 | 202,252 |
| Bergen | 5 | — | 5 | — | — | — | 110,424 |
| Hordaland | 2 | — | 2 | 1 | — | 1 | 188,389 |
| Sogn-Fjordane | — | — | — | — | 2 | 2 | 96,849 |
| Möre-Romsdal | 8 | — | 8 | 2 | 1 | 3 | 182,859 |
| S. Tröndelag (including Trondheim) | 7 | — | 7 | 2 | 1 | 3 | 193,912 |
| N. Tröndelag | 1 | — | 1 | — | — | — | 105,679 |
| Nordland | 6 | — | 6 | — | 4 | 4 | 215,972 |
| Troms | 3 | — | 3 | — | 0 | 0 | 113,722 |
| Finnmark | 4 | — | 4 | 1 | 3 | 4 | 58,790 |
| Sea-faring | 2 | — | 2 | 1 | — | 1 | — |
| Unknown | 3 | — | 3 | 1 | 1 | 2 | — |
| | 172 | 4 | 176 | 30 | 29 | 59 | |

* Part of greater Oslo, including important industry, was formerly situated in Akershus fylke and the figures on population presented in this table refer to conditions before the inclusion of these areas in Oslo of to-day.

not before an autopsy has been performed. There is no intended initial selection of surgically favourable histological types from the remote parts of the country. Actually, for patients from the remote parts of the country the histological typing is very rarely done before the patient has entered the larger hospital for treatment. If a selection has taken place, this may have been decided by the general physical state and the age of the patient. Admittedly, some of the Group II tumours (especially the benign adenomas) may afflict younger persons and present a longer case-history, and thereby cause a certain selection, but others, like the bronchiolar cell carcinomas and the adenocarcinomas do not differ essentially in their clinical development from the Group I tumours.

If now Table II is examined, it may be permissible to conclude that Group I and Group II tumours differ considerably and systematically in their pattern of occurrence throughout the country. Considering the small number, it seems that the occurrence of the Group II tumours are of the same order of magnitude over the whole country. The number of cases in Oslo, the main centre of diagnostic facilities, is proportionately not higher than in the other counties, and the counties in the extreme north are well represented. The figures seem to confirm our previous conclusion that this group of tumours is caused by factors of a general nature, at least factors striking males and females living under the most different social conditions, with equal force (Kreyberg, 1954*a*). They should represent an important part of what Clemmesen, Nielsen and Jensen (1953) call " unavoidable " cancers and Lickint (1953) calls " die gen-gebundenen Formen " (the genedetermined types). Their uniform appearance in the present material greatly strengthen our view that the material really is representative for all Norway.

Turning to the Group I tumours, a very different picture is found. Oslo has a prominent position, with a very high number of Group I tumours. Next in order of frequency come Östfold, Vestfold and Telemark, counties which are characterized by a considerable accumulation of industrial towns, and centres with urbanized living but without the administrative status of towns.

Some of the other counties are more difficult to analyse, because of the small figures and the rather mixed social conditions, but it is worth noting that the lowest figures for Group I tumours are found in the counties with the highest representation of agriculture, forestry and fisheries, (Hedmark, Opland, Aust-Agder), in spite of the fact that some of these counties, like Opland and Hedmark, have excellent hospitals and are in easy communication with Oslo. The low total figures for both histological groups from Bergen and the surrounding counties point to a lagging co-operation from that part of the country, as, according to the clinical figures of Myhre (1953), lung cancer actually is occurring in an appreciable number. The counties of the extreme north show higher figures for Group I tumours than Hedmark and Opland, in spite of the marked longer distance to the diagnostic centres.

In order to elucidate this question further, the patients have been classified according to the type of community, where they had their life and work, in one of the following groups: (i) as living in the country; (ii) in rural or non-industrial towns; (iii) in smaller industrial towns, as well as in industrial centres without the status of town; or (iv) in larger towns, the latter comprising Oslo, Bergen and Trondheim.

The separation of industrial centres with no status of towns from rural districts, where they administratively belong in our official statistics, is logical in this respect, because life in such places is urbanized and the air is more or less polluted as well, sometimes to a degree sufficient to cause severe damage to plant life in the vicinity. Among such places may be mentioned Sauda, Odda, Årdal, etc.

In Table III the data for these primary localisations of the patients are given. In the table, ratios for Group I : Group II tumours, and males : females are inserted, not only for the primary geographical localisations, but also for combinations of such. The combinations have been directed at (1) the elucidation of the relative influence of a domicile in areas with " clean air " versus areas with " air polluted by smoke and fumes from industry", and (2) the lung cancer occurrence in areas with strictly " rural mode of life " on one hand (countryside) compared to areas with a more, or less, marked " urban mode of life " on the other. This latter group comprises larger and smaller towns plus industrial centres without the status of towns.

It is evident, that a strict distinction between towns with " clean air " and towns with " polluted air" is impossible, and that the classification is based upon a personal judgment, which can easily be questioned. Likewise, the separation of Bergen and Trondheim as larger towns may be disputed. For this reason, the figures for Oslo, the only really large town in Norway, have been given separately.

There is, however, one feature, which indicates that the differences reported upon are real, namely the ratios Group I tumours : Group II tumours, as well as the ratios males : females, because these ratios are based upon comparable figures.

TABLE III.—*Distribution of Group I and Group II Lung Tumours in Urban and Rural Areas.*

| | Group I. | | | Group II. | | | Ratio. | |
|---|---|---|---|---|---|---|---|---|
| | M. | F. | Total. | M. | F. | Total. | I : II. | M. : F |
| (i) Countryside | 25 | 1 | 26 | 13 | 15 | 28 | 0·9 : 1 | 2·2 : 1 |
| (ii) Non-industrial towns | 38 | 1 | 39 | 4 | 5 | 9 | 4·3 : 1 | 7·0 : 1 |
| (iii) Industrial towns and centres | 20 | — | 20 | 5 | 3 | 8 | 2·5 : 1 | 8·3 : 1 |
| (iv) Larger towns (Oslo, Bergen, Trondheim) | 68 | 2 | 70 | 2 | 5 | 7 | 10·0 : 1 | 10·0 : 1 |
| Varied. Unknown | 3 | — | 3 | 1 | 1 | 2 | | |
| Areas with "clean air" (No. (i) and (ii)) | 63 | 2 | 65 | 17 | 20 | 37 | 1·7 : 1 | 3·5 : 1 |
| Areas with "polluted air" (No. (iii) and (iv)) | 88 | 2 | 90 | 7 | 8 | 15 | 6·0 : 1 | 9·5 : 1 |
| Areas with "rural life" (No. (i)) | 25 | 1 | 26 | 13 | 15 | 28 | 0·9 : 1 | 2·2 : 1 |
| Areas with "urban life" (No. (ii), (iii) and (iv)) | 126 | 3 | 129 | 11 | 13 | 24 | 5·4 : 1 | 8·6 : 1 |
| Smaller towns (No. (ii) and (iii)) | 58 | 1 | 59. | 9 | 8 | 17 | 3·5 : 1 | 7·4 : 1 |
| Oslo | 59 | 2 | 61 | 1 | 4 | 5 | 12·2 : 1 | 10·0 : 1 |
| Sea | 18 | — | 18 | 5 | — | 5 | 3·6 : 1 | — |

Finally, sailors have been treated as a separate group for two reasons. Firstly, they do not belong to any of the other classes of domicile. This explains some differences in the figures for Oslo, for instance, in Tables II and III. Secondly, they have no female partners living under similar circumstances.

With these reservations the following conclusions have been drawn:

The findings in Table III strengthen our previous assumption from Table II that the different occurrence of Group I and Group II tumours is systematic.

In strictly rural districts the ratio between the two groups of tumours, as well as between the two sexes mirror the situation for the whole country, and even for Oslo, a few decades ago (Jackobsen, 1953 ; Christiansen, 1953).

The larger towns, with Oslo definitely leading, show the most marked deviations, with a very strong rise in the Group I tumours.

An analysis of the figures for the smaller towns and industrial centres shows that all urban areas take part in the development, and these results are in complete conformity with the findings from other countries.

An interesting point is that the smaller non-industrial towns, with "clean air", and often situated in rural surroundings show a development in line with the capital and the other towns, not lagging behind the other smaller towns.

These findings indicate that the new carcinogenic situation is closely linked to the urban mode of life, and that smoke and fumes from industry is not the essential factor of that part of urban life which is responsible for the increase in lung cancer in Norway.

### SUMMARY AND CONCLUSIONS.

The new carcinogenic situation established in Norway approximately at the time of the first world war, and manifesting itself in the middle of the nineteen-forties by a marked rise in Group I tumours in males (Kreyberg, 1954*b*), has in the present paper been analysed as to its local geographic distribution.

On the basis of 235 cases from the whole country it has been shown that the new development is not yet perceptible in true rural districts, which still show the same distribution of the different histological types and the same sex ratio as presented by the country total and also by Oslo a few decades ago.

The new development is, on the other hand, definitely established in all types of urban settlements. Oslo shows a leading position, with the smaller towns slightly behind, including industrial centres with no formal standing as towns. Smaller non-industrial towns, many in rural surroundings, do not show lower figures than other small towns. These findings lead to the conclusion that the new carcinogenic situation is closely linked to the urban mode of life, and that smoke and fumes from industry are not an essential factor of that part of urban life which is responsible for the increase in lung cancer in Norway.

The present study has been aided by a generous grant from " Tobaksfabrikernes Landsforening av 1901".

I wish to express my sincere thanks to Dr. Knut Westlund for valuable advice during the preparation of this paper.

### REFERENCES.

Christiansen, T.—(1953) *Brit. J. Cancer*, **7**, 248.

Clemmesen, J., Nielsen, A., and Jensen, E.—(1953) *Acta Un. int. Cancr.*, **8**, Fasc. Spec., 160.

Jakobsen, A.—(1953) *Brit. J. Cancer*, **7**, 432.

Kreyberg, L.—(1952) *Ibid.*, **6**, 112.—(1954*a*) *Ibid.*, **8**, 199.—(1954*b*), *Ibid.*, **8**, 209.

Lickint, F.—(1953) 'Ätiologie und Prophylaxe des Lungenkrebses.' Dresden und Leipzig (Steinkopf).

Myhre, J.—(1953) *Tidsskr. norske Lægeforen*, **73**, 802.